# Retinoblastoma protein regulates carcinogen susceptibility at heterochromatic cancer driver loci

**DOI:** 10.26508/lsa.202101134

**Published:** 2022-01-04

**Authors:** Ka Man Wong, Devin A King, Erin K Schwartz, Rafael E Herrera, Ashby J Morrison

**Affiliations:** Department of Biology, Stanford University, Stanford, CA, USA

## Abstract

Carcinogenic insult, such as UV light exposure, creates DNA lesions that evolve into mutations if left unrepaired. These resulting mutations can contribute to carcinogenesis and drive malignant phenotypes. Susceptibility to carcinogens (i.e., the propensity to form a carcinogen-induced DNA lesion) is regulated by both genetic and epigenetic factors. Importantly, carcinogen susceptibility is a critical contributor to cancer mutagenesis. It is known that mutations can be prevented by tumor suppressor regulation of DNA damage response pathways; however, their roles carcinogen susceptibility have not yet been reported. In this study, we reveal that the retinoblastoma (RB1) tumor suppressor regulates UV susceptibility across broad regions of the genome. In particular, centromere and telomere-proximal regions exhibit significant increases in UV lesion susceptibility when *RB1* is deleted. Several cancer-related genes are located within genomic regions of increased susceptibility, including telomerase reverse transcriptase, *TERT*, thereby accelerating mutagenic potential in cancers with RB1 pathway alterations. These findings reveal novel genome stability mechanisms of a tumor suppressor and uncover new pathways to accumulate mutations during cancer evolution.

## Abstract (precis)

The retinoblastoma protein regulates mutagenic potential via control of UV-induced DNA lesion acquisition, thus revealing novel mechanisms that contribute to its tumor suppressor capabilities.

## Introduction

Loss of tumor suppressor function is a fundamental characteristic of malignancy. Tumor suppressors regulate proliferation, senescence, and apoptotic pathways through a variety of mechanisms that sense extracellular environments and intracellular stress signals (Hanahan & Weinberg, 2011). The first identified tumor suppressor was the retinoblastoma gene, *RB1*, which causes pediatric malignancy of the eye after biallelic inactivation (Fung et al, 1987; Lee et al, 1987; Horowitz et al, 1989).

RB1 regulates cell cycle progression through its repression of E2F-responsive genes (Weinberg, 1995). It is active in early G1 phase and inactivated via phosphorylation by CDKs during the G1 to S transition to allow expression of genes, such as S-phase cyclins, that promote DNA synthesis and cell growth. CDK inhibitors *CDKN2A*/p16 and *CDKN1A*/p21 can also inhibit phosphorylation of RB1 by binding to and inactivating CDKs, thereby preventing cell cycle progression. This elaborate tumor suppressor-regulated network restricts cell cycle growth and proliferation to favorable environments that promote survival (Weinberg, 1995).

RB1 functions as a transcriptional repressor of cell cycle genes through its association with chromatin modifiers, which effectively inhibits target gene expression (Dick & Rubin, 2013). Specifically, RB1 associates with histone deacetylases, H3K9 and H3K27 methyltransferases, and heterochromatin protein 1 (HP1) to alter the chromatin around target loci (Luo et al, 1998; Nielsen et al, 2001; Morrison et al, 2002; Ishak et al, 2016). Interestingly, RB1 has also been found to regulate constitutive heterochromatin, particularly at centromeres and telomeres, impacting chromatid cohesion and telomere length regulation (García-Cao et al, 2002; Gonzalo et al, 2005; Manning et al, 2010, 2014; Ishak et al, 2016; Gonzalez-Vasconcellos et al, 2017).

Alteration of the RB1 pathway is pervasive in many sporadic cancers (Dyson, 2016). For example, the RB1 pathway is a primary driver of skin cancers, with mutations in *RB1*, *CDKN2A*/p16, *CDKN1A*/p21, *CDKN1B*/p27, *CDKN2B*/p15, *CCNE1*/cyclin E, and *CCND1*/cyclin D, collectively, in 26.4% of melanomas (Hayward et al, 2017). Loss of tumor suppressor function allows unrestricted proliferation and accumulation of genome instabilities that promote malignant transformation (Hanahan & Weinberg, 2011). This transformation can, expectedly, be accelerated in the presence of carcinogens that initiate mutagenesis. Indeed, alteration of the RB1 pathway in mice leads to increases in tumor formation, particularly after exposure to chemical carcinogens and radiation (Serrano et al, 1996; Krimpenfort et al, 2001; Sharpless et al, 2001). For example, *CDKN2A*/p16 deletion leads to increased metastatic melanoma after carcinogen exposure (Krimpenfort et al, 2001).

Cancers initiated by carcinogen exposure, such as skin and lung cancers, are among those with the highest frequency of somatic mutation (Alexandrov et al, 2013). Melanoma, in particular, is caused by UV exposure in sunlight, the most ubiquitous environmental carcinogen. The major classes of DNA lesions induced by UV radiation are cyclobutane pyrimidine dimers (CPDs) and 6–4 photoproducts (6–4PPs) (Douki & Cadet, 2001), which are targeted by nucleotide excision repair pathways, transcription-coupled repair and global genomic repair (Hanawalt & Spivak, 2008). CPDs are the most abundant UV-induced DNA lesion and proposed to be the most mutagenic (You et al, 2001; Jans et al, 2005; Pfeifer & Besaratinia, 2012; Brash, 2015). Interestingly, the distribution of UV-induced mutations is not uniform across melanoma genomes and found to be regulated by regional features surrounding mutation sites, such as base composition and replication timing (Wolfe et al, 1989; Stamatoyannopoulos et al, 2009). Additionally, transcriptionally repressive heterochromatic regions accumulate more mutations than transcriptionally active euchromatin (Schuster-Böckler & Lehner, 2012; Polak et al, 2015), which is contributed by lack of transcription-coupled repair in non-genic regions and inefficient global genomic repair in heterochromatic regions (Hu et al, 2015, 2017).

However, mutation frequency can also be influenced by regional propensity to form UV-induced DNA lesions (i.e., susceptibility), which precedes repair. Formation of these UV-induced DNA lesions is the initial step of mutagenesis, and thus, essential for establishing mutagenic potential throughout the genome. Interestingly, published data from our laboratory demonstrates that epigenomic features, such as repeat-rich heterochromatin, influence the abundance of ultraviolet CPD lesion formation across the genomes of primary cells (García-Nieto et al, 2017). In these assays, cells were exposed to brief (less than 10 s) dose of UV light then immediately lysed and purified DNA for immunoprecipitation (IP) with CPD-specific antibodies. Repair of CPD lesions is marginally detectable (<5% of all lesions) within 1 h after UV exposure (Moser et al, 2005; Verbruggen et al, 2014; Adar et al, 2016), thus these assays are designed to measure acute UV-mediated DNA lesion formation rather than repair kinetics.

Surprisingly, we found that DNase I–hypersensitive, gene-dense regions with active transcription, are among the chromatin states that are the least UV susceptible (García-Nieto et al, 2017). Conversely, DNase I–protected heterochromatin with repressed transcription is more susceptible to UV compared with the genome-wide average. Importantly, these trends in UV susceptibility are still observed when corrected for dipyrimidine frequency. Thus, susceptibility is not solely driven by base composition, but can be regulated by epigenetic features. Notably, UV susceptibility in primary cells closely reflects mutation frequency across the genomes of malignant melanomas, thus susceptibility is a significant contributor to mutagenesis during cancer evolution.

Because of its roles in chromatin regulation and tumor suppression, we investigated whether RB1 could also influence UV susceptibility, a mechanism distinct from its previously characterized roles. Given that heterochromatin features positive correlate with UV susceptibility (García-Nieto et al, 2017), we proposed that loss of RB1-regulated heterochromatin would reduce acquisition of UV lesions in corresponding regions of the genome. However, we found that deregulation of heterochromatin at and around telomeres and centromeres in *RB1* knockout cells correlates with increased susceptibility. Notably, several cancer driver genes are located in these regions of increased susceptibility, such as *TERT*, thereby increasing its mutagenic potential. Correspondingly, *TERT* mutations are significant elevated in melanomas with RB1 pathway alterations.

These results uncover new mechanisms that regulate carcinogen susceptibility. Importantly, this study reveals novel pathways in which tumor suppressors can promote genome stability, and how these pathways can become disrupted to accelerate mutagenesis and malignancy.

## Results

**Figure 1.**
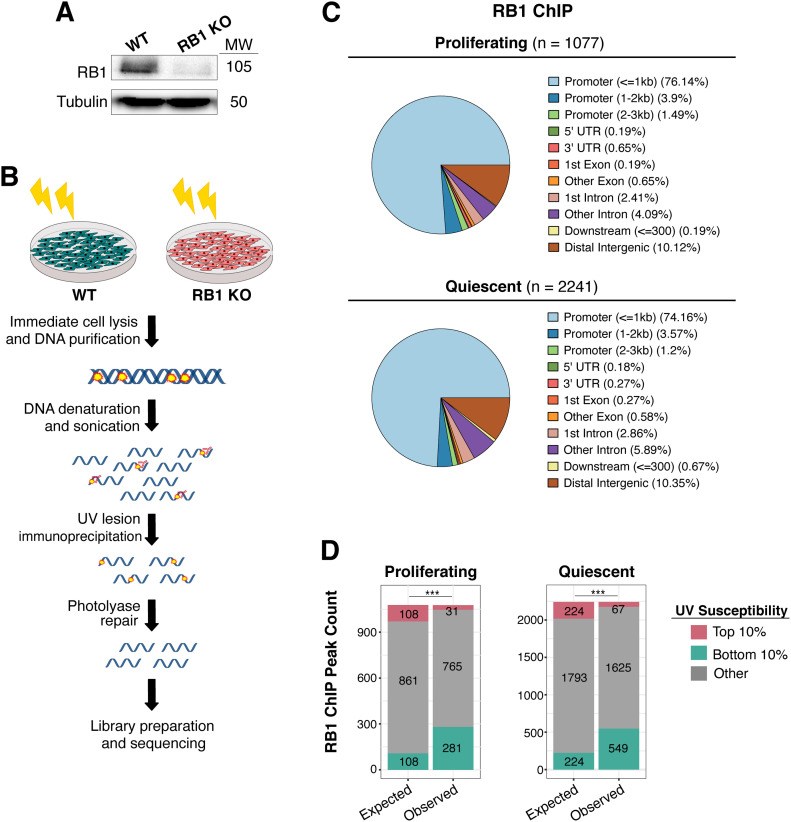
Deletion of *RB1* alters UV susceptibility. **(A)** Western blot of RB1 in *RB1* knockout (RB1 KO) and wild-type (WT) IMR-90 cells. Tubulin was used as loading control. Molecular weight (MW) in kD is shown next to blot. **(B)** Illustration of UV lesion mapping method. Both wild-type and RB1 KO cells were treated with 100 J/m^2^ UVC. Cells were immediately lysed after UV irradiation and DNA was harvested. Purified DNA was then immunoprecipitation with anti-cyclobutane pyrimidine dimer antibody. Immunoprecipitated fragments were then repaired by photolyases prior to library preparation and Illumina sequencing. **(C)** Annotation of previously published RB1 peaks in proliferating and quiescent IMR-90 cells (Chicas et al, 2010). **(D)** Expected versus observed RB1 ChIP-seq peaks in top 10% and bottom 10% UV susceptible genomic regions, in both proliferating and quiescent cells. Expected values were generated based on the null hypothesis that susceptibility change has no association with location of RB1 peaks. Chi-squared test (*P* < 0.001) indicated that there is significant depletion and enrichment of RB1 peaks in top 10% and bottom 10% regions, respectively.

After creation of *RB1* knockout (hereafter referred to as RB1 KO) via CRISPR-Cas9 editing in IMR90 primary cells (Fig 1A), the CPD lesion IP protocol was repeated as previously described (García-Nieto et al, 2017) and shown in Fig 1B. Importantly, before UV exposure, cells were confluent for 2 d to synchronize cells in G1 phase of the cell cycle and avoid cell cycle effects on susceptibility resulting from reduced RB1 function. FACS analysis determined comparable cell cycle occupancy for both cell lines, specifically 79% G1, 4% S-phase, and 18% G2/M for wild-type; and 81% G1, 3% S-phase, and 16% G2/M for RB1 KO cells.

Differences in UV susceptibility between wild-type and RB1 KO cells were first examined at previously published RB1 ChIP-seq binding sites throughout the genome (Chicas et al, 2010). Indicative of its role as a transcription factor of cell cycle genes, RB1 was found to be enriched at promoters in both proliferating (76%) and quiescent (74%) cells (Fig 1C). For both these conditions, the majority of these RB1 binding sites did not overlap with the genomic regions that have significant changes in UV-induced lesions in RB1 KO cells compared with wild-type (Fig 1D). However, there is an enrichment of a minor number of RB1 binding sites in the least (bottom 10%) UV susceptible regions of the genome, and a corresponding reduction of overlap in the most (top 10%) susceptible regions. This is in agreement with previous results demonstrating that gene-rich regions, including those regulated by RB1, are among the most protected against UV-induced damage (García-Nieto et al, 2017); and loss of RB1 function does not significantly increase UV susceptibility in these genic regions.

### RB1 regulates UV susceptibility in heterochromatin regions

However, as previously introduced, RB1 not only regulates cell cycle genes but also heterochromatin features at centromeres (Gonzalo et al, 2005; Isaac et al, 2006; Manning et al, 2010) and telomeres (García-Cao et al, 2002). Specifically, loss of RB1 function is associated with changes in the distribution of both constitutive H3K9me3 and facultative H3K27me3 heterochromatin modifications in these regions (Ishak et al, 2016; Gonzalez-Vasconcellos et al, 2017).

**Figure 2.**
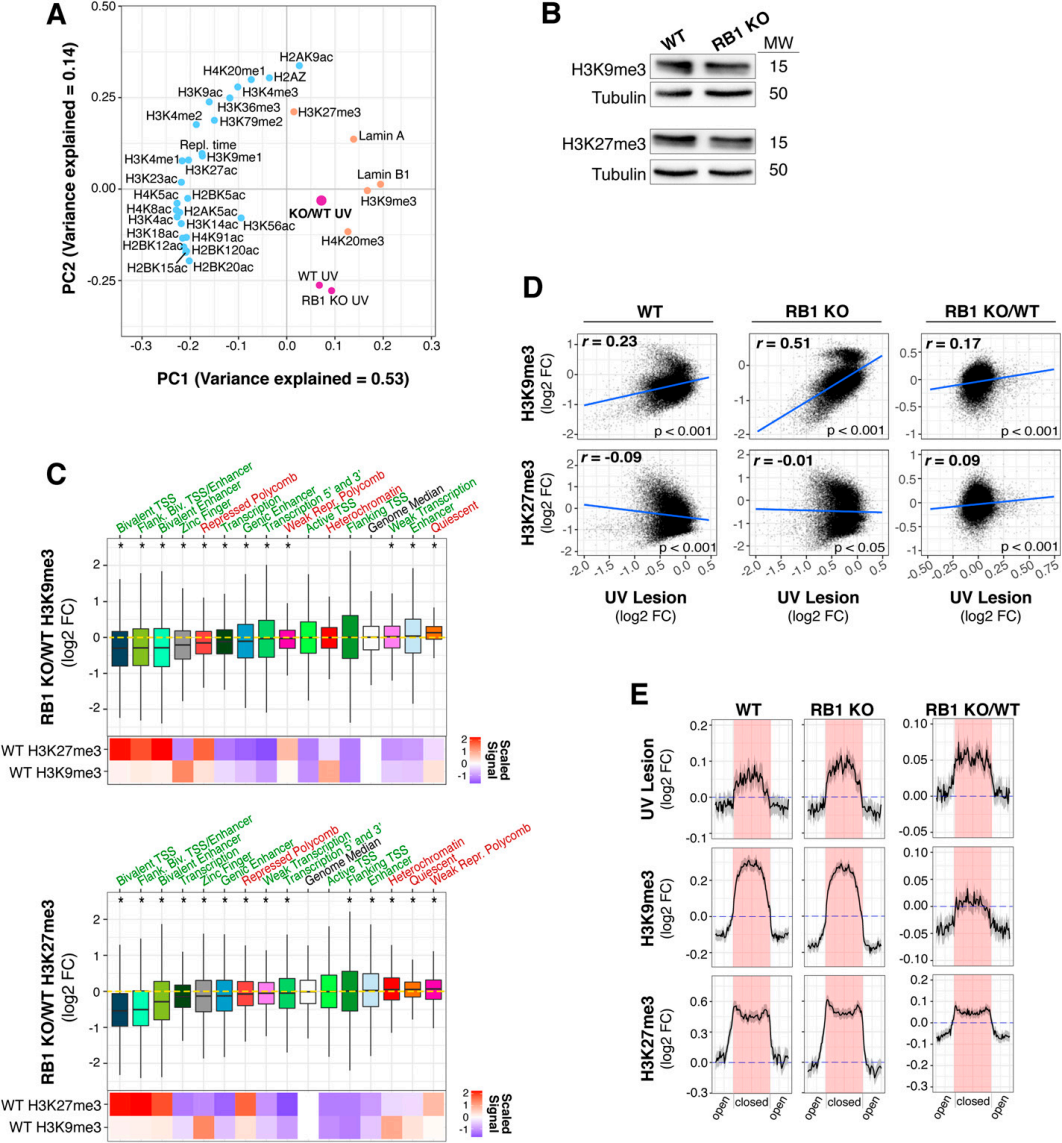
Genome-wide susceptibility changes in RB1 KO cells are associated with heterochromatin alterations. **(A)** Principal component analysis of epigenetic features, replication timing, and UV lesions (UV) in 100 kb bins genome-wide (The ENCODE Project Consortium, 2012; Sadaie et al, 2013; Lund et al, 2014; Roadmap Epigenomics Consortium et al, 2015; Nelson et al, 2016). WT, wild-type; KO, RB1 KO. Variance explained is labelled for principal component 1 (PC1) and PC2. Known euchromatic features are labelled with blue dots, heterochromatic with orange dots, and UV lesions with purple dots. **(B)** Western blot of H3K9me3 and H3K27me3 in *RB1* knockout (RB1 KO) and wild-type (WT) IMR90 cells. Tubulin was used as loading control. Molecular weight (MW) in kD is shown next to blot. **(C)** Box plots of log_2_ fold change (FC) of histone modifications (H3K9me3 and H3K27me3) in RB1 KO compared with WT (RB1 KO/WT) in 15 chromatin states from IMR90, as defined in the Roadmap Epigenomics Consortium et al (2015). TSS, transcriptional start site; Biv., Bivalent; Poly., Polycomb. Known heterochromatic states are labelled in red, euchromatic states are labelled in green. Genome-wide median is labelled by the white box and horizontal yellow dotted line. Statistical outliers were omitted. Mann–Whitney *U* test denotes significantly different (*P* < 0.001) states from the genome median, indicated by *. Heat maps below box plot depict scaled signal of median H3K9me3 and H3K27me3 ChIP signal in each chromatin state. **(D)** Scatter plot (100 kb bins) with spearman correlation of log_2_ FC UV lesion and H3K9me3 and H3K27me3 histone modifications in RB1 KO, WT, and RB1 KO versus WT (RB1 KO/WT). Linear regression line is shown in blue. **(E)** UV lesion, H3K9me3 and H3K27me3 levels in WT A and B Hi-C compartments, previously determined (Fortin & Hansen, 2015), in RB1 KO, WT, and FC of RB1 KO versus WT (RB1 KO/WT). Each compartment was divided into 50 bins for signal calculation. **(A, B)** The closed compartments (B) shaded in red, were aligned in the middle with the adjacent open compartments (A) on each side. The mean signal and 95% confidence interval via bootstrapping (R = 1,000) are in black and grey, respectively.

Because UV susceptibility is mechanistically linked to H3K9me3-enriched heterochromatin (García-Nieto et al, 2017), the epigenetic features associated with changes in susceptibility upon RB1 loss were explored. Specifically, UV lesion abundances between wild-type and RB1 KO cells were examined in relation to a variety of histone modifications mapped by the Roadmap Epigenomics Project (Roadmap Epigenomics Consortium et al, 2015). Principal component analysis of genome-wide UV lesion abundances in both wild-type and RB1 KO cells cluster closely with constitutive heterochromatin features, such as H3K9me3 and lamin B1, followed by H3K27me3-enriched chromatin, whereas most euchromatic modifications do not cluster with UV susceptibility (Fig 2A).

H3K9me3 and H3K27me3 ChIP-seq was then performed in wild-type and RB1 KO cells to identify chromatin changes that may contribute to altered susceptibility. At a cellular level, no significant differences in these histone marks were observed (Fig 2B). However, compared with wild-type cells, RB1 KO cells exhibit reduced H3K9me3 in several bivalent and zinc finger chromatin states (Fig 2C). These bivalent states are enriched in H3K27me3 and H3K9me3 in wild-type cells (Bernstein et al, 2006; Matsumura et al, 2015), yet in RB1 KO cells, levels were reduced. Conversely, more subtle, yet statistically significant, increases in these heterochromatin marks were identified in the “Quiescent” state for H3K9me3 and “Weak Repressed Polycomb” for H3K27me3. A reduction of repressive marks in RB1 KO cells is consistent with RB1’s association with repressive histone modifiers (Dick & Rubin, 2013), and alterations in bivalent domains is in agreement with the association of RB1 with the EZH2 repressor complex that mediates bivalent domain silencing (Ishak et al, 2016).

Across the genomes of wild-type cells we found a positive correlation between UV susceptibility and H3K9me3, whereas H3K27me3 exhibited a modest negative correlation (Fig 2D, and as previously reported [García-Nieto et al, 2017]). The relationship between susceptibility and H3K9me3 and, to a lesser extent, H3K27me3 was reinforced in RB1 KO cells (Fig 2D).

Interestingly, the link between heterochromatic features and UV susceptibility is strong enough to be observed at the level of topologically associated domains (TADs) composed of insulated heterochromatin and euchromatin compartments, and corresponding histone marks and transcriptional activity (Dixon et al, 2016). Specifically, the heterochromatic compartments of TADs acquire more UV lesions than euchromatic compartments, with a sharp transition between the two (Fig 2E). Notably, in RB1 KO cells this pattern of UV susceptibility is exaggerated with heterochromatic compartments accumulating even more UV lesions, and euchromatic compartments less, than wild-type cells. Euchromatic TAD compartments with decreased susceptibility in RB1 KO cells exhibited corresponding decreases in heterochromatin marks. However, in this analysis, increases in UV susceptibility did not correlate with elevated H3K9me3.

The results in Fig 2 demonstrate that RB1 regulates heterochromatin features, which often correlate with UV susceptibility. In particular, decreases in heterochromatin marks in genic regions correspond to reductions in UV susceptibility. However, increases in susceptibility do not obviously appear to be linked to elevated constitutive heterochromatin features in RB1 KO cells.

### Loss of *RB1* results in elevated UV susceptibility at centromeric and telomeric regions

**Figure 3.**
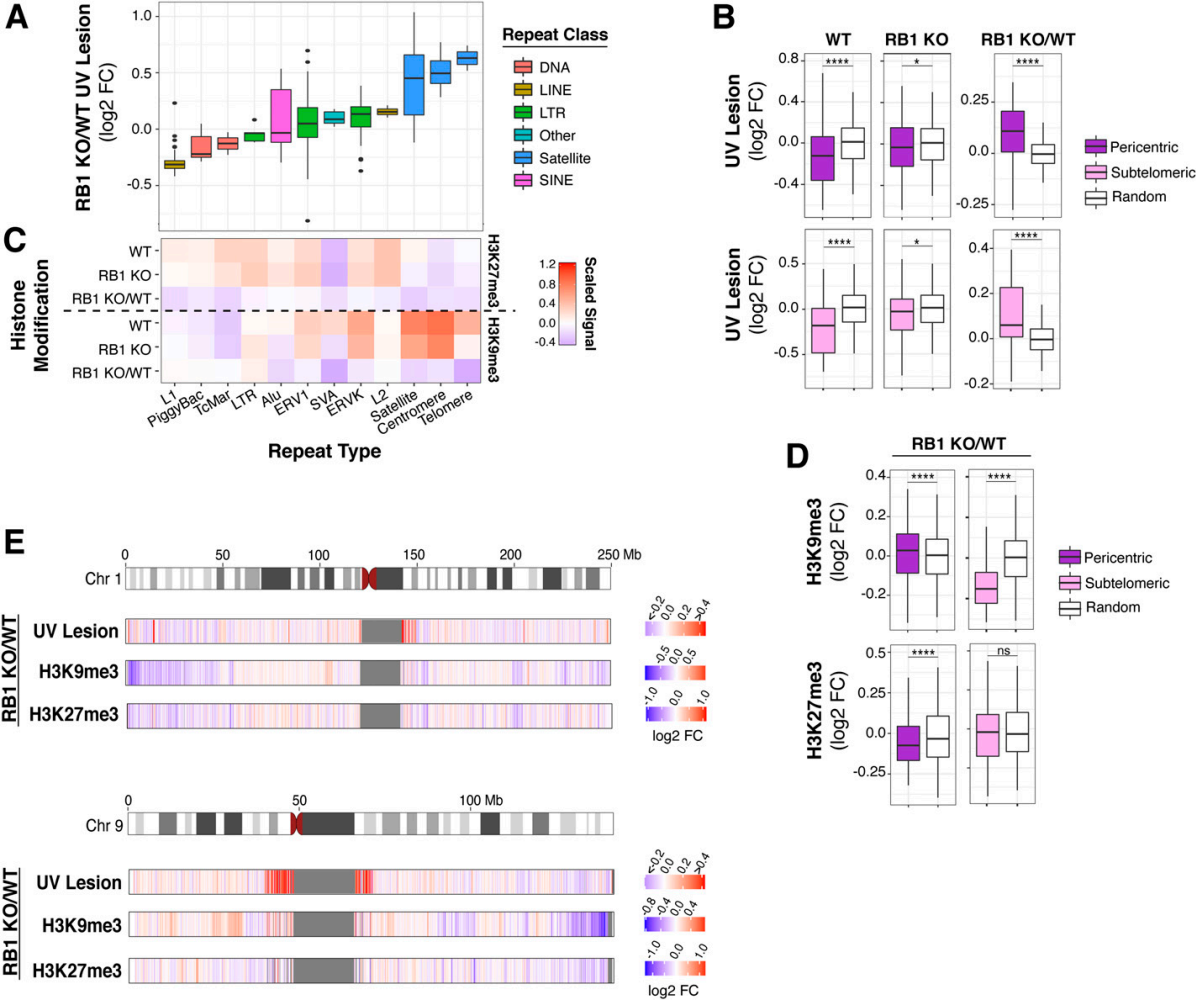
Centromeric and telomeric regions are more susceptible to UV following *RB1* knockout. **(A)** Box plots of UV lesion log_2_ fold change (FC) in RB1 KO cells compared with wild-type (WT) (RB1 KO/WT) in selected families of repeat sequences. **(B)** Box plots of UV lesion abundance in pericentric or subtelomeric regions, defined as 1 Mb bins next to centromeres or 100 kb next to telomeres, in RB1 KO, WT, and RB1 KO/WT FC. Mann–Whitney *U* test indicates significant difference between 1,000 groups of random regions and selected regions (ns, not significant, *P < 0.05, **P < 0.01, ***P < 0.001, ****P < 0.0001). **(C)** Heat map of scaled median H3K9me3 and H3K27me3 ChIP signal for RB1 KO, WT, and RB1 KO/WT FC in selected families of repeat sequences. For each histone modification, the signal is normalized to genome median for visualization. **(D)** Box plots of H3K9me3 and H3K27me3 ChIP signal in pericentric and subtelomeric regions, for RB1 KO, WT, and RB1 KO/WT FC. Mann–Whitney *U* test indicates significant difference between 1,000 groups of random regions and selected regions (ns, not significant, *P < 0.05, **P < 0.01, ***P < 0.001, ****P < 0.0001). **(E)** Heat maps of RB1 KO/WT log_2_ FCs in UV lesion, H3K9me3, and H3K27me3 signal in 100-kb bins along selected chromosomes.

**Figure S1.**
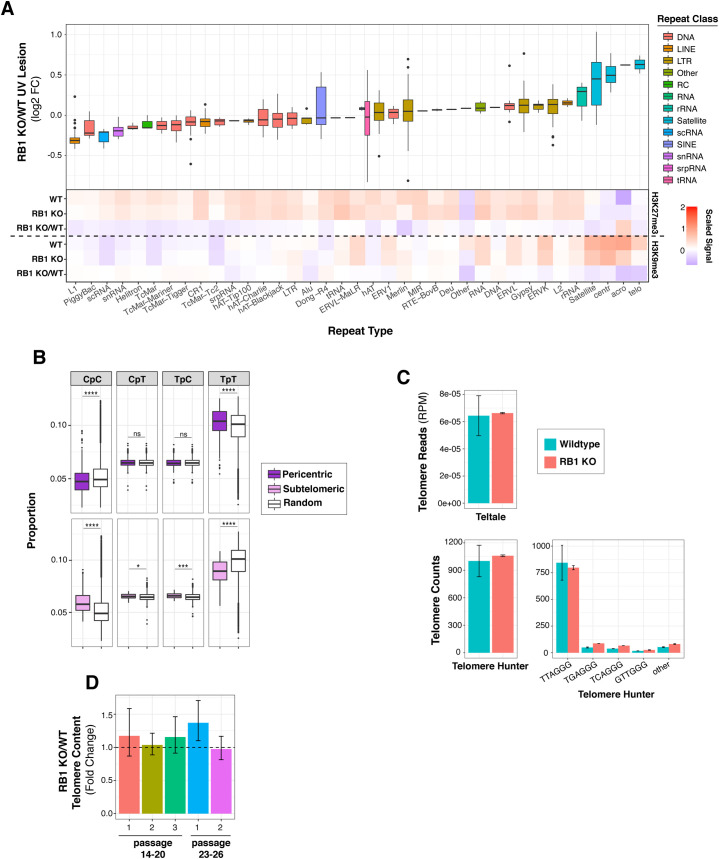
RB1 regulates UV susceptibility in repeat sequences. **(A)** Top, box plots of UV lesion log_2_ fold change (FC) in RB1 KO cells compared with wild type (WT) (RB1 KO/WT) in selected families of repeat sequences. Bottom, heat map of scaled median H3K9me3 and H3K27me3 ChIP signal for RB1 KO, WT, and RB1 KO/WT FC in selected families of repeat sequences. For each histone modification, the signal is normalized to genome median for visualization. **(B)** Dipyrimidine content of subtelomeric and pericentric regions, defined as 1 Mb bins next to centromeres or 100 kb next to telomeres, compared to random genome control. Mann–Whitney *U* test indicates significant difference between 1,000 groups of random regions and selected regions (ns, not significant, *P < 0.05, **P < 0.01, ***P < 0.001, ****P < 0.0001). **(C)** Abundance of telomere sequences in RB1 KO cells compared to WT using Teltale and Telomere Hunter, as described in Materials and Methods section. **(D)** Fold change differences in telomere content between WT and RB1 KO cells quantified by qPCR. Cells were sampled at indicated passage number. Two to three biological replicates, and three technical replicates for each biological replicate, were used. Standard error was calculated using error propagation.

Because of RB1’s role in modulating the chromatin of centromeric and telomeric repeat regions, UV susceptibility was then investigated in an extensive panel of repeat sequences. Interestingly, a large variation of susceptibility was observed in several repeats (Figs 3A and S1A). Telomeric, centromeric, and satellite repeats, which are enriched around centromeres, exhibited significant increases in susceptibility, whereas L1 repeats exhibited decreased susceptibility.

Notably, in wild-type cells, centromeric and telomeric-proximal regions are relatively protected from UV compared with a random genomic control (Fig 3B). This protection is particularly unexpected for pericentric regions as TpT frequency, the dipyrimidine with the highest propensity to form a CPD (Douki & Cadet, 2001), is elevated in these regions compared with a random control (Fig S1B). However, in RB1 KO cells, this observed protection for both centromeric and telomeric-proximal regions is significantly disrupted, resulting in an overall increase in UV susceptibility in RB1 KO compared with wild-type (Fig 3B). Notably, increases in telomere length in RB1 KO cells were not observed (Fig S1C and D), thus are not attributed to additional telomeric sequences in mutant cells.

**Figure S2.**
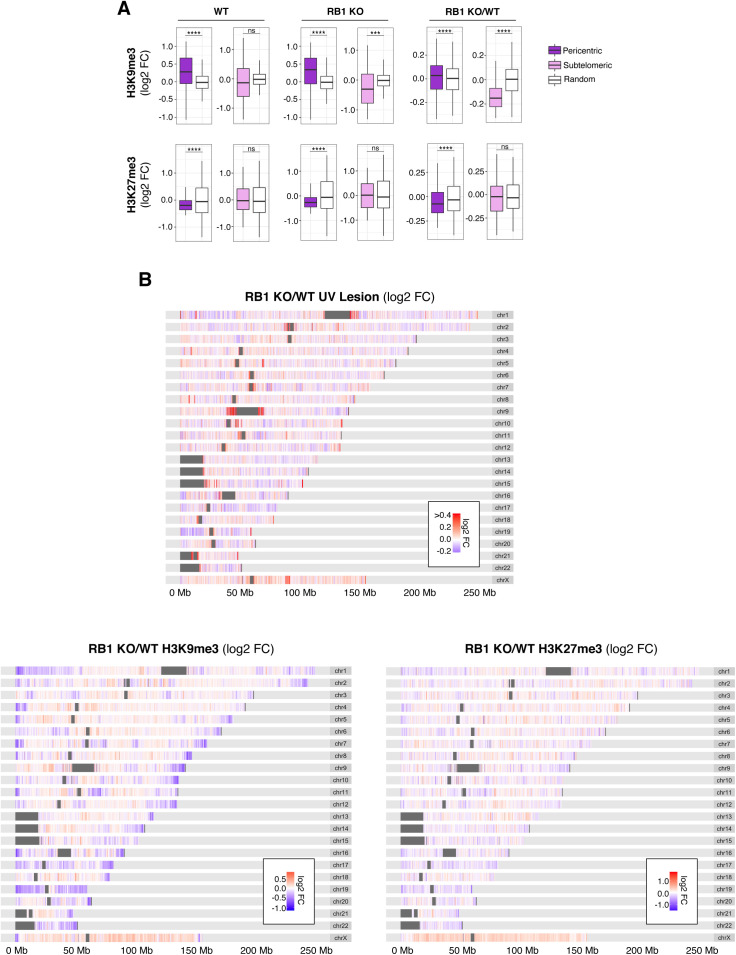
RB1 regulates chromatin and UV susceptibility in centromeric and telomeric regions. **(A)** Box plots of H3K9me3 and H3K27me3 ChIP signal in pericentric and subtelomeric regions, for RB1 KO, wild-type (WT), and RB1 KO/WT fold change (FC). Mann–Whitney *U* test indicates significant difference between 1,000 groups of random regions and selected regions (ns, not significant, *P < 0.05, **P < 0.01, ***P < 0.001, ****P < 0.0001). **(B)** Heat maps of RB1 KO/WT log_2_ FC in UV lesion, H3K9me3, and H3K27me3 signal in 100 kb bins along autosomal chromosomes.

In wild-type cells, these repeat sequences are relatively enriched in heterochromatic H3K9me3 and/or H3K27me3 marks (Figs 3C and S1A). However, in RB1 KO cells, there is a general decrease in these repressive marks at and around these repeats (Fig 3C and D). Some repeats exhibit less H3K27me3, such as L1, whereas centromeric and telomeric repeats exhibit decreases in both H3K27me3 and H3K9me3 (Figs 3C and S1A). Pericentric and subtelomeric regions also display alterations H3K27me3 and/or H3K9me3 (Figs 3D and S2A). Interestingly, these changes in heterochromatin features and UV susceptibility around centromeric and telomeric regions can be observed at a chromosomal level (Figs 3E and S2B). At several telomeric and centromeric regions, changes in H3K9me3 and increases in UV susceptibility can be clearly observed, whereas alterations in H3K27me3 are relatively more dispersed throughout the chromosome.

Thus, these repeat-focused analyses differ from the genome-wide overviews presented in Fig 2 and demonstrate that whereas heterochromatin alterations at telomeric and centromeric regions can lead to altered susceptibility, the directionality is not conserved.

### Expression of centromere-associated genes is altered after *RB1* knockout

Previous studies demonstrate that loss of RB1 and RB family members results in decondensation of telomeres and centromeres, reduced localization of specialized proteins, such as condensin, and chromosomal abnormalities (García-Cao et al, 2002; Gonzalo et al, 2005; Isaac et al, 2006; Coschi et al, 2010, 2014; Longworth & Dyson, 2010; Manning et al, 2010; van Harn et al, 2010). In the case of telomeres specifically, previous studies demonstrate their sensitivity to UV-induced lesion formation (Kruk et al, 1995; Rochette & Brash, 2010), which can persist for days to weeks in cell culture (Mitchell et al, 1999; Bérubé et al, 2018). Interestingly, telomere-associated proteins, such as the Shelterin complex (de Lange, 2005), have been shown to regulate UV susceptibility (Parikh et al, 2015), demonstrating the potential for non-histone, DNA-associated proteins to regulate acquisition of DNA lesions.

**Figure 4.**
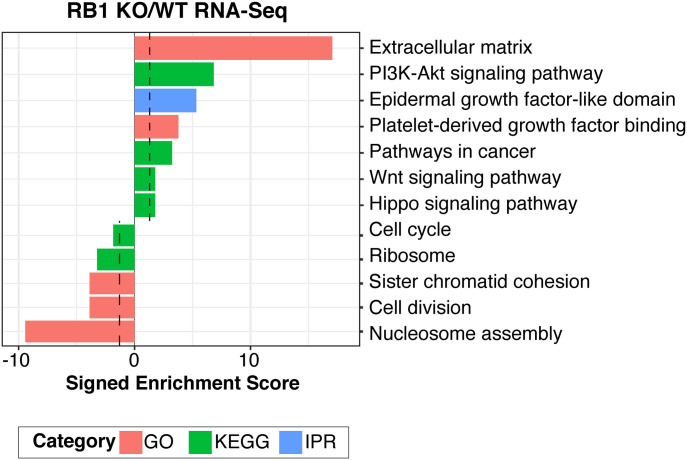
RB1 regulates genes in mitotic pathways. DAVID functional enrichment analysis (Huang et al, 2009a, 2009b) of significantly differentially expressed genes (upregulated n = 471; downregulated n = 180) from RNA-seq in RB1 KO cells compared to wild-type. Signed enrichment score (−log_10_ *P*-value) is displayed for each functional group, with the significance cutoff (*P* = 0.05) in black. GO, gene ontology; KEGG, Kyoto Encyclopedia of Genes and Genomes; IPR, InterPro.

To better characterize UV susceptibility defects associated with centromeres and telomeres in RB1 KO cells, RNA-seq was performed to detect changes in the expression of genes associated with centromeres and telomeres (Table S1). Functional annotation analysis of significantly differentially expressed genes identified several down-regulated categories, including “nucleosome assembly,” “cell division,” “chromatid cohesion,” and “kinetochore” (Fig 4), with specific centrosome-associated proteins (CENP) being down-regulated. Up-regulated categories include the “PI3K-Akt signaling pathway,” “pathways in cancer,” and “extracellular matrix” (Fig 4). These results, together with previous publications demonstrating loss of proper centromeric formation upon RB1 depletion (Gonzalo et al, 2005; Isaac et al, 2006; Coschi et al, 2010; Manning et al, 2010), strongly suggest that RB1 regulates centromere assembly to both ensure proper mitotic chromosome function and protect against UV damage formation.

Table S1. Functional enrichment categories of RB1-regulated genes.

In the case of telomeric regions specifically, expression of related genes, such as Shelterin, were not significantly altered in RB1 KO cells (Table S1). However, previous studies demonstrate that telomeric heterochromatin is regulated by RB1 and related family members (García-Cao et al, 2002; Gonzalo et al, 2005; Gonzalez-Vasconcellos et al, 2017). Thus, general telomere dysfunction, which can alter association of telomere-binding proteins, may be linked to alteration of UV susceptibility.

### RB1-regulated pericentric and telomeric regions are more mutable in cancer

**Figure 5.**
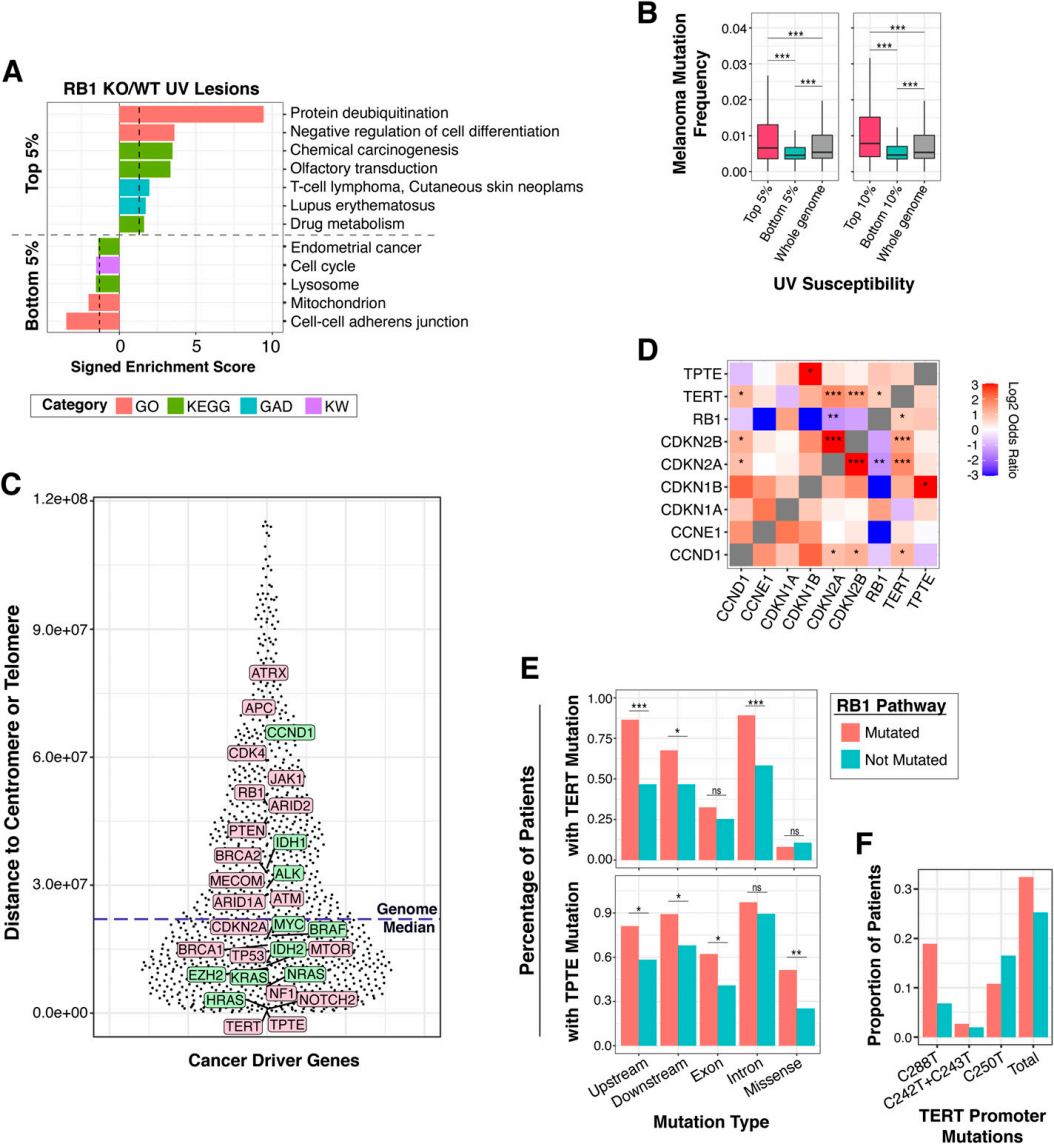
Regions of RB1-regulated susceptibility include highly mutated cancer driver genes. **(A)** DAVID functional enrichment analysis (Huang et al, 2009a, 2009b) of genes in the top and bottom 5% UV susceptible regions (top n = 821; bottom n = 2,600) in RB1 KO cells compared with wild-type. Only genes with >10% overlap in top or bottom bin are included in analysis. Signed enrichment score (−log_10_ *P*-value) is displayed for each functional group, with the significance cutoff line (*P* = 0.05) in black. GO, gene ontology; KEGG, Kyoto Encyclopedia of Genes and Genomes; GAD, Genetic Association Database; KW, Keyword. **(B)** Box plots of melanoma mutation frequency (Hoadley et al, 2018) of genes in the top and bottom 10% and 5% susceptible regions compared with the whole genome. Only C>T and G>A mutation were counted. Mutation rates were normalized by cytosine and guanine content of the gene. Additional details provided in the Materials and Methods section. Mann–Whitney *U* test indicates significant melanoma mutation frequency difference between groups (ns, not significant, *P < 0.05, **P < 0.01, ***P < 0.001). **(C)** Distance of cancer driver genes to the closest centromere or telomere in base pairs. Genome median is shown as blue dotted line in graph. Genes with oncogenic function are shown in green and genes with tumor suppressor function are shown in pink. **(D)** Mutational co-occurrence (in red) and mutual exclusivity (in blue) of genes in RB1 pathway, *TERT* and *TPTE*. Log_2_ odds ratio analysis was performed as described in the Materials and Methods section (*q < 0.05, **q < 0.01, ***q < 0.001). **(D, E)** Percentage of melanoma patients with *TERT* and *TPTE* mutations within different genic features from patients with (mutated) and without (not mutated) RB1 pathway mutations for genes shown in (D). Only C>T and G>A mutation were counted. Mutation rates were normalized by cytosine and guanine content of the gene. Additional details provided in the Materials and Methods section (ns, not significant, *P < 0.05, **P < 0.01, ***P < 0.001). **(D, F)** Proportion of *TERT* promoter mutations at different ETS sites from patients with (mutated) and without (not mutated) RB1 pathway mutations for genes shown in (D). Source data are available online for this figure.

Because elevated susceptibility can increase mutagenic potential, we assessed mutation burden in regions of the genome that acquire more UV-induced lesions in RB1 KO cells compared with wild-type. Functional annotation analysis of the genes within the most susceptible (top 5%) genomic regions in RB1 KO cells identified cancer and disease-related categories, including “Chemical Carcinogenesis” and “T-cell lymphoma and Cutaneous Skin Neoplasms” (Fig 5A and Table S2). Categories representing genes in the least susceptible (bottom 5%) genomic regions include several important for essential cell functions, such as “Mitochondrion” and “Lysosome.” “Cell cycle” is also represented, supporting results of Figs 1 and 2, which show that RB1-regulated genes have reduced UV susceptibility and heterochromatin formation. “Endometrial Cancer” is also identified as representing the least susceptible regions of the genome and may indicate some degree of tissue-specific carcinogen susceptibility.

Table S2. Functional enrichment analysis of differentially susceptible genes in RB1 KO cells.

**Figure S3.**
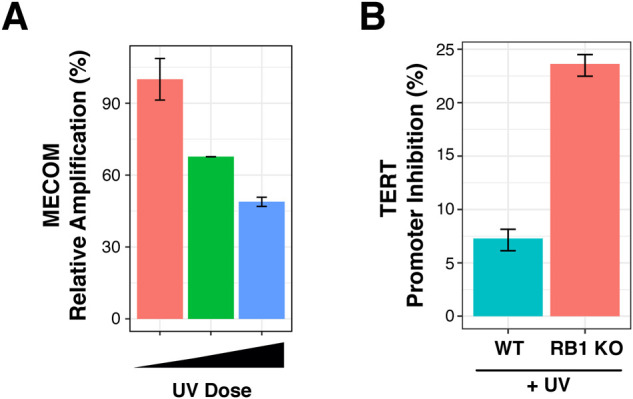
Knockout of RB1 increases UV susceptibility at the *TERT* promoter. **(A)** Dose dependent inhibition of qPCR amplification of *MECOM* locus, a highly susceptible locus, in wild-type (WT) cells (García-Nieto et al, 2017). Relative qPCR amplification is compared with *GAPDH*, a relatively protected locus in WT cells. UV doses are 0, 100, 200 J/m^2^ UVB. **(B)** qPCR inhibition of the *TERT* promoter in both RB1 KO and WT cells after 100 J/m^2^ UVC exposure. Primers amplify −224 to −308 of *TERT* promoter and includes ETS sequences. Three technical replicates were used for each sample. Standard error was calculated using error propagation.

Interestingly, genes in the most susceptible regions have significantly increased mutation rates in melanoma compared with the least susceptible (Fig 5B). Notably, a few cancer driver genes are found within these regions (Table S2). Among them is telomerase reverse transcriptase (*TERT*), which is widely mutated in a majority of cancers and ∼10% of melanoma (Hoadley et al, 2018). Somatic mutations in the *TERT* promoter create de novo ETS binding sites to up-regulate *TERT* expression during immortalization (Horn et al, 2013; Huang et al, 2013; Chiba et al, 2017). Several of these mutations carry the C>T transition indicative of UV-induced mutagenesis, also known as “solar signature” mutation (Brash, 2015). The increased susceptibility of the *TERT* promoter was also confirmed using a qPCR interference assay and found to be approximated threefold more in RB1 KO cells compared with wild-type (Fig S3A and B).

Another cancer-associated gene found to have increased UV susceptibility in RB1 KO cells, and mutation rates in melanoma, is *TPTE* (Table S2). *TPTE* is a relatively uncharacterized PTEN-related tyrosine phosphatase that is mutated in 26% of cutaneous melanoma (Hoadley et al, 2018). Interestingly, compared with the genome median, several of these cancer driver genes are located either proximal to a centromere or telomere (Fig 5C). *TERT* is proximal to the telomere of chromosome 5p, whereas *TPTE* is proximal to the centromere of chromosome 21p (Fig 5C).

**Figure S4.**
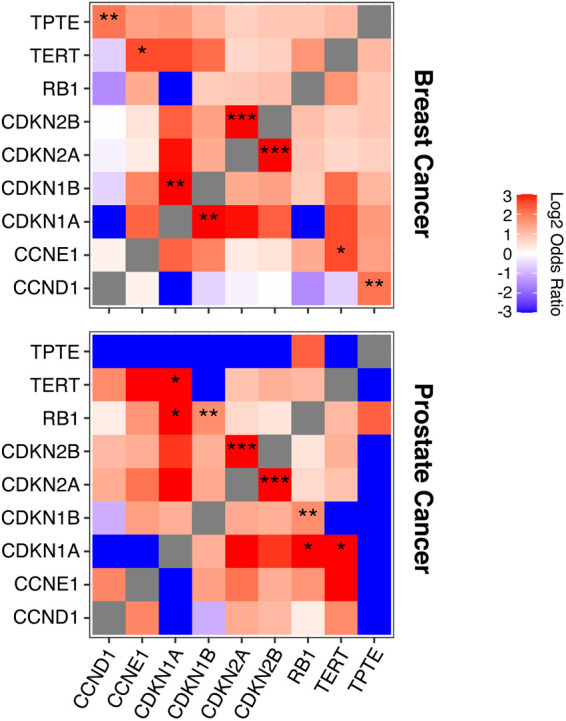
Mutational co-occurrence of *TERT*, *TPTE*, and the RB1 pathway in breast and prostate cancer. Mutational co-occurrence (in red) and mutual exclusivity (in blue) of genes in RB1 pathway, *TERT* and *TPTE* in breast and prostate cancer. Log_2_ odds ratio analysis was performed as described in the Materials and Methods section (*q < 0.05, **q < 0.01, ***q < 0.001).

Using sequencing datasets from 1,970 melanomas, we found that cancers with RB1 pathway mutations, including *CDKN2A*/*p16*, have significant co-occurrence of mutations in *TERT* and *TPTE* (Fig 5D). Mutational co-occurrence between these genes was less prominent in cancers not known to be caused by carcinogen exposure, such as breast and prostate cancer (Fig S4). Further analyses into the specific mutated regions of *TERT* identified upstream promoter, downstream, and intronic regions as having increased mutation frequency in melanomas that also carry mutations in the RB1 pathway compared with tumors without RB1 pathway alterations (Fig 5E). Site-specific ETS mutations were also found to be differentially enriched in tumors with RB1 pathway mutations (Fig 5F). Significant increases in *TPTE* mutations were also found in the promoter, downstream, and exonic regions (Fig 5E).

**Figure S5.**
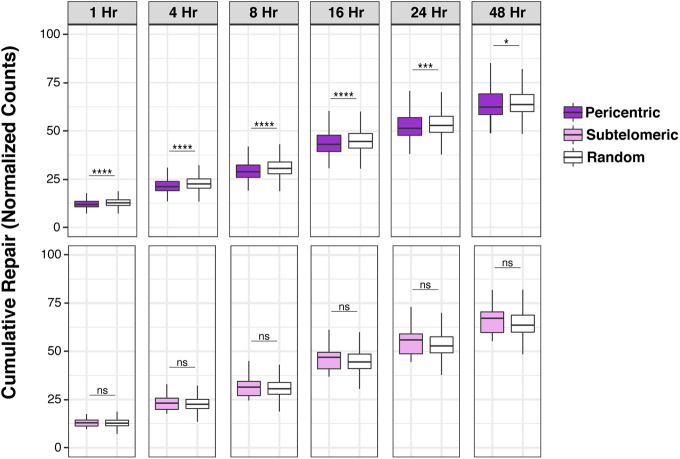
Repair rates of pericentric and subtelomeric regions. Cumulative XR-seq repair rate at pericentric regions and subtelomeric regions (Hu et al, 2017), compared with random genome control. Mann–Whitney *U* test indicates significant difference between 1,000 groups of random regions and selected regions (ns, not significant, *P < 0.05, **P < 0.01, ***P < 0.001, ****P < 0.0001).

These results show that regions of increased UV susceptibility resulting from *RB1* loss contain several highly mutated cancer driver genes located proximal to heterochromatic telomeres and centromeres. These increases in mutation frequency can be compounded by reduced repair efficiency, which is observed in pericentric, but not subtelomeric, regions of wild-type cells (Fig S5).

## Discussion

Collectively, these results demonstrate for the first time that disruption of a tumor suppressor can increase susceptibility to carcinogenic insult, thus accelerating mutagenic potential. This is a significant expansion of our current understanding of tumor suppressors, which are largely known to combat mutagenesis through their participation in DNA damage response pathways (Hanahan & Weinberg, 2011). Specifically, these results demonstrate that tumor suppressors can influence genome maintenance via regulation of carcinogen susceptibility.

Importantly, *RB1* loss increases opportunities for mutation of several cancer driver genes, most notably the *TERT* gene, which plays critical roles in carcinogenesis by sustaining proliferative capacity (Maciejowski & de Lange, 2017). *TERT* is proximal to a telomere, which, along with centromeric regions, is among the most UV susceptible in RB1 KO cells. This is in line with previous findings demonstrating the important role of RB1 in regulating the proper function and formation of both telomeres and centromeres (García-Cao et al, 2002; Gonzalo et al, 2005; Isaac et al, 2006; Coschi et al, 2010, 2014; Longworth & Dyson, 2010; Manning et al, 2010; van Harn et al, 2010). As expected, increased UV lesions and mutation of centromeric and telomeric sequences can also significantly impair chromosome segregation and increase chromosomal fusions (Lin et al, 2004; Wright & Shay, 2005; Finkel et al, 2007). Indeed, loss of *RB1* results in aneuploidy in both retinoblastoma patient-derived fibroblasts and mouse models (Coschi et al, 2014).

Mutagenic potential is, of course, contributed by both susceptibility and repair. Previous studies demonstrate that loss of *RB1* alters UV DNA damage responses (Bosco, 2005). Specifically, whereas checkpoint responses are abrogated in mutant cells, repair of UV-induced photoproducts is accelerated. It is hypothesized that this combination of events could lead to propagation of mutations by reducing opportunities for apoptosis if errors are incorporated during accelerated repair. Interestingly, hereditary retinoblastoma patients have increased risk for melanoma development (Moll et al, 1997; Fletcher et al, 2004). Elevated frequency of carcinogen-induced secondary cancers, such as melanoma, in retinoblastoma patients may, indeed, be contributed by both increased susceptibility and altered repair responses.

The specific mechanisms of how RB1 protects telomeric and centromeric regions against carcinogenic insult are still under investigation. Interestingly, although whole genome patterns of UV susceptibility in RB1 KO cells agree with previous results and demonstrate that heterochromatin features positively associate with increased UV susceptibility (García-Nieto et al, 2017), it was not a feature consistent with susceptibility at centromeres and telomeres. Nevertheless, we find that expression of centromere binding proteins, such as CENP proteins, are reduced in RB1 KO cells. In addition, condensin binding to centromeres is disrupted in cells lacking RB1 function (Coschi et al, 2014; Marshall et al, 2019). Collectively, reduced binding of centromere-associated proteins likely contributes to both centromere dysfunction, as well as increased UV susceptibility in RB1 KO cells.

Notably, non-histone DNA binding proteins have previously been shown to regulate UV susceptibility. Namely CTCF and ETS transcription factors alter UV lesion formation at their binding sites (Mao et al, 2016; Fredriksson et al, 2017; Hu et al, 2017). TRF1 of the telomere binding Shelterin complex also protects against UV lesion formation (Parikh et al, 2015). Shelterin provides a protein scaffold that promotes significant DNA compaction important for telomere protection (Bandaria et al, 2016). In addition, heterochromatin plays important roles in the regulation of telomere compaction. Specifically, loss of heterochromatic H3K9me3 in telomeres is associated with decreased heterochromatin protein 1 (HP1) association (García-Cao et al, 2002), which, in turn, impairs telomere cohesion and length maintenance (Canudas et al, 2011). Indeed, H3K9me3 is reduced in telomeric regions of RB1 KO cells, thus binding of heterochromatin proteins would be impaired, which could render associated DNA sequences more vulnerable to UV damage.

These results suggest that disruption of local chromatin structure can alter binding of chromatin-associated proteins that regulate UV susceptibility. These findings have important consequences on genome stability mechanisms that can become disrupted during cancer evolution. Many cancer drivers that are also chromatin regulators, such as RB1, have broad impacts across the genome (Gonzalo et al, 2005; Longworth & Dyson, 2010; Plass et al, 2013; Helin & Minucci, 2017; Valencia & Kadoch, 2019). One specific example is oncogenic Myc overexpression, which is common in cancers, and alters histone modifications globally (Knoepfler et al, 2006; Cotterman et al, 2008; Varlakhanova & Knoepfler, 2009). These global changes in chromatin structure likely impact the association of many DNA binding proteins, which ultimately can alter susceptibility to carcinogens and accelerate mutagenic potential in several regions of the genome.

Findings in this report demonstrate that loss of tumor suppressor function can accelerate mutagenesis via novel genome stability mechanisms that do not involve damage response pathways. These results provide a foundation for additional research on the regulation of carcinogen susceptibility and its disruption in diseases fueled by mutation, such as cancer.

## Materials and Methods

### Cell culture

Human fetal lung fibroblast IMR90 (Cat. no. CCL-186; ATCC) were obtained from ATCC. Cells were cultured at 37°C and 5% CO_2_ in DMEM with high glucose, sodium pyruvate, and L-glutamine (Cat. no. SH3024301; Hyclone), supplemented with 15% (vol/vol) FBS (Cat. no. S11150; Atlanta Biologicals) and 1% Pen/Strep (Cat. no. SV30010; Hyclone). HEK293T (Cat. no. CRL-3216; ATCC) cells were obtained from ATCC. Cells were cultured at 37°C and 5% CO_2_ in DMEM with high glucose, sodium pyruvate, and L-glutamine, supplemented with 10% (vol/vol) FBS and 1% Pen/Strep.

### CRISPR-Cas9 knockout cell creation

Guide RNA was selected and designed to target *RB1* translational start site using the Broad design tool (Doench et al, 2016; Sanson et al, 2018) (guide RNA sequence: 5′-CACCGGCATGACGCCTTTCCGCGGC-3′ 3′-CCGTACTGCGGAAAGGCGCCGCAAA-5′) and cloned into lentiCRISPR plasmid (Cat. no. 49535; Addgene) and confirmed by Sanger sequencing. The construct was transfected into HEK293T cells with VSV-g, psPAX2 to produce virus to transduce IMR-90 cells. Puromycin resistant cells were pooled together. *RB1* disruption was confirmed by Western blot with anti-RB1 antibody (4H1, Cat. no. 9309; Cell Signaling Technology).

### CPD IP, library preparation, and sequencing

Wild-type and RB1 KO IMR90 cells were grown to full confluency to eliminate cell cycle effects on UV lesion acquisition. CPD IP was performed as previously described (García-Nieto et al, 2017). Cells were irradiated with 100 J/m^2^ UVC and were immediately lysed with 1% SDS buffer. Cell lysates were treated with RNase A (Cat. no. NC9729931; Thermo Fisher Scientific) and proteinase K (Cat. no. P2308; Sigma-Aldrich), followed by DNA precipitation with ethanol/sodium acetate. Purified DNA was then sonicated by a Bioruptor (Diagenode) and heated to 99°C to denature. The resulting single-stranded fragments were incubated with anti-CPD antibody (clone TDM-2; Cosmos Bio). Immunoprecipitated DNA fragments were repaired by CPD and 6-4PP photolyases (Selby & Sancar, 2012). Library preparation was performed with NEBNext Ultra II Directional RNA Second Strand Synthesis Module (Cat. no. E7550; NEB) and NEBNext ChIP-seq Library Prep Master Mix Set for Illumina (Cat. no. E6240; NEB) as instructed by the manufacturer. Paired-end sequencing was performed on HiSeq 4000 with read length of 100 bp.

### Western blotting

Cells were lysed by preheating lysis buffer (1% SDS and 25 mM Tris, pH 8.0) to 99°C and adding to cells. Lysates were then spun down at >18,000*g* and supernatants were separated on PAGE gels and transferred to polyvinylidene difluoride (PVDF) membranes (Cat. no. 1620177; Bio-Rad) at 100 V at room temperature for 1 h. The membrane was then blocked with 5% BSA (Cat. no. A-420; GoldBio) for 1 h at room temperature and incubated with primary antibody overnight at 4°C. Antibodies used are as follows: anti-H3K9me3, Cat. no. 8898; Abcam; anti-H3K27me3, Cat. no. 6002; Abcam; anti-tubulin Cat. no. sc-53030; Santa Cruz Biotechnology; anti-retinoblastoma 1, Cat. no. 9309; Cell Signaling Technology. The membrane was then incubated with peroxidase-coupled secondary antibody for 1 h at room temperature (donkey anti-rabbit IgG-HRP, Cat. no. NA-934; Cytiva; sheep anti-mouse IgG-HRP, Cat. no. NA-931; Cytiva; goat anti-rat IgG-HRP, NB7115; Novus Biological). Chemiluminescent signal was detected with Advansta Westernbright Quantum-HRP (K-12042). Tubulin was used as loading control.

### Histone chromatin IP (ChIP), library preparation, and sequencing

∼2 × 10^6^ confluent cells were fixed with 1% formaldehyde (Cat. no. BP-531; Thermo Fisher Scientific) for 5 min at room temperature under gentle agitation. Formaldehyde was then quenched with the final concentration of 0.125 M glycine. Cells were then washed with ice-cold PBS twice, and washed twice in micrococcal nuclease (MNase) buffer (50 mM Tris–HCl, pH 8.0, 250 mM sucrose, 25 mM KCI, 1 mM MgCl_2_, 1 mM CaCl_2_, 0.5 mM 2-mercaptoethanol, and 0.1% NP-40). Nuclei were isolated and DNA was digested with 400 gel units of MNase (Cat. no. M0247; NEB) for 16 min on ice, and digestion was terminated with MNase stop buffer (1% SDS, 50 mM Tris–HCl, pH 8.0, and 20 mM EGTA). Nuclei were then sonicated for 2 min on low with a bioruptor (30 s ON/OFF; Diagenode). Cell debris was removed by centrifugation at 20,000*g* for 15 min at 4°C. DNA fragment size was verified via bioanalyzer and most fragments (∼90%) were the size of mono or di-nucleosomes (<500 bp). The supernatant was diluted with RIPA buffer without SDS, and 5% of the sample was used as the input for the experiment.

It is important to note that we performed MNase digestion rather than sonication as a method to fragment heterochromatin. This is because when sonication was performed, the immunoprecipitated heterochromatin fragments were greater than 2 kb, whereas total input chromatin displayed a mean fragment size of 250 bp. We reasoned that heterochromatin does not adequately fragment when using sonication. If sonication were used, the vast majority of immunoprecipitated DNA would not be represented in sequencing reads, which requires less than 1 kb DNA fragments.

Antibodies (H3K9me3, Cat. no. 8898; Abcam; H3K27me3, Cat. no. 6002; Abcam, Rabbit IgG, Cat. no. 2729; Cell Signaling Technology) were pre-coupled to Protein G Dynabead (Cat. no. 10-0040D; Thermo Fisher Scientific) in RIPA buffer before IP with the sample. Coupled beads were blocked with 0.1 mg/ml BSA (Fraction V, sterile filtered) for 1 h. Samples were then incubated with antibody-coupled beads overnight at 4°C, followed by elution in elution buffer (1× TE with 1% SDS) at 30°C for 15 min. Eluted DNA–protein complexes were then treated with 10 mg of RNAse A at 37°C for 1 h and proteinase K treatment at 37°C for 2 h. Samples were de-crosslinked overnight at 65°C. De-Crosslinked DNA was purified using Chromatin IP DNA Purification Kit (Cat. no. 58002; Active Motif). Library preparation was performed using NEBNext Ultra II DNA Library Prep Kit for Illumina (Cat. no. E7645S; NEB) and NEBNext Multiplex Oligos for Illumina (Cat. no. 6440S; NEB) as instructed by the manufacturer. Paired-end sequencing was performed on HiSeq 4000 with read length of 100 bp.

### ChIP-sequencing data processing

Sequencing data were processed according to ENCODE (phase-3) specification for ChIP-seq, using the ENCODE Transcription Factor and Histone ChIP-Seq processing pipeline (The ENCODE Project Consortium, 2012) (github: https://github.com/ENCODE-DCC/chip-seq-pipeline2). In short, reads were aligned to the reference genome hg19 using Bowtie2 (Langmead & Salzberg, 2012). Aligned reads were then filtered and deduplicated based on their quality. Signal tracks of fold change were generated using MACS2.0 (Zhang et al, 2008) by normalizing IP signals by the input.

### RNA-sequencing and analysis

RNA was isolated from confluent cells using Quick-RNA Miniprep plus kit (Cat. no. T1057; Zymo Research). rRNA was then removed using Ribo-Zero Magnetic Kit (Cat. no. 20040526; Illumina), followed by library preparation using NEBNext Ultra II Directional RNA Library Prep Kit for Illumina (Cat. no. E7760S; NEB). RNA quality was examined on Agilent 2100 and all samples had RIN (RNA integrity number) of 10. At least 40 million paired-end reads with read length of 150 bp were sequenced per replicate on an Illumina NovaSeq 6000.

Raw sequencing reads were aligned to GrCh37 with gencode.v19.annotation.gtf using STAR with the following arguments: --readFilesCommand gunzip --outSAMtype BAM SortedByCoordinate --outSAMattributes Standard --quantMode GeneCounts --outFilterMultimapNmax 1 --outFilterMatchNmin 35 --twopassMode Basic (Dobin et al, 2013). Output quantification file was used as input to DESeq2 to identify differentially expressed genes, using cutoff padj = 0.1 (Love et al, 2014).

### Repetitive sequence analysis

Raw sequencing reads are aligned to hg19 with Bowtie2 (Langmead & Salzberg, 2012) with default settings. PCR duplicates were then removed with picard MarkDuplicates with --REMOVE_DUPLICATES=TRUE. De-duplicated bam files were then used as input of RepEnrich2 (Criscione et al, 2014) (github: https://github.com/nerettilab/RepEnrich2) to quantify reads aligned to each type of repetitive sequence as annotated by RepeatMasker. Briefly, RepEnrich2 builds a custom genome repeat reference using both the canonical sequence of each repeat and the annotated repetitive sequence of each repeat in RepeatMasker (Smit et al, 2013–2015). Each repeat is assigned to a specific repeat class through unique alignment. Reads that align to multiple families of repeats are divided amongst families.

Repeat enrichment analysis was performed using quantification as input of DESeq2 (Love et al, 2014). For histone modifications, input from RB1 KO and wild-type have different distributions of repeats. Therefore, DESeq2 was used to find significantly different repeats in both input and ChIP. Output log_2_ fold change value was used to compare the distribution of ChIP versus input via Wilcoxon paired test for each group. Permutation test was performed to assess how likely the difference is obtained by chance, and the result was corrected for multiple testing using Benjamini–Hochberg.

### Centromere and telomere analysis

Coordinates of centromeres and telomeres of hg19 were obtained from UCSC genome browser (table browser; assembly: Feb. 2009 (GRCh37/hg19), group = All Tracks, track = gap). Pericentric and subtelomeric regions are defined as the ±1 Mb region around the centromere and 100 kb region proximal to the telomere, respectively. Binned genomic regions of 100 kb that overlapped with the defined pericentric or subtelomeric regions were included in the analysis. The same number of regions were selected at random as control. Mann–Whitney *U* test indicates significant difference between 1,000 groups of random regions and selected regions.

### TAD analysis

A/B compartments of IMR-90 derived from Hi-C were obtained from Fortin and Hansen (2015). Adjacent compartments of the same type were merged to create a dense track for A/B compartments. Each compartment was binned into 50 bins and the median signal for each bin was calculated. The closed domains, shaded in red, were aligned in the middle with its flanking open domains on the side. The mean signal and 95% confidence interval obtained via bootstrapping (R = 1,000) is in black and grey, respectively.

### Melanoma mutation frequency analysis

Simple somatic mutation data for melanomas were downloaded from the International Cancer Genome Consortium Data Coordination Centre (Hayward et al, 2017) (https://dcc.icgc.org/). Acral melanoma that are not caused by UV exposure were discarded. Donors were separated into two groups based on their mutation status of RB1-pathway genes (including *RB1*, *CCND1*, *CCNE1*, *CDKN1A*, *CDKN1B*, *CDKN2A*, and *CDKN2B*). For genic mutational frequency analysis, only C>T and G>A mutation were counted. Mutation rates were normalized by cytosine and guanine content of the gene. Significant differences in mutation rates were detected via Willcox-test.

A set of consolidated cancer driver genes (n = 1,152) was compiled as the union of lists of cancer driver genes from the Catalog of Somatic Mutations (COSMIC) Cancer Gene Census (Tate et al, 2019), the PanCancer Driver Gene list (Bailey et al, 2018), the IntOGen cancer driver database (Gonzalez-Perez et al, 2013), and novel cancer driver genes identified by MutPanning (Dietlein et al, 2020).

### Mutation co-occurrence and exclusivity analysis

Eight non-overlapping melanoma studies (n = 1,970 tumors) from cBioPortal for Cancer Genomics were included in this analysis (Berger et al, 2012; Hodis et al, 2012; Krauthammer et al, 2012; Snyder et al, 2014; Van Allen et al, 2014; Akbani et al, 2015). The analysis was performed with the in-house tools of cBioPortal (Cerami et al, 2012; Gao et al, 2013). Briefly, odds ratio tests were performed to identify any mutually exclusive or co-occurring events. Fisher’s exact test was then performed to evaluate the significance of identified events. Two prostate adenocarcinoma studies (n = 2,478) and four breast cancer studies (n = 1,376) from cBioPortal for Cancer Genomics were also included in this analysis (The Oslo Breast Cancer Consortium (OSBREAC) et al, 2012; Banerji et al, 2012; Shah et al, 2012; PCF/SU2C International Prostate Cancer Dream Team et al, 2018; Nguyen et al, 2020). Three additional data sets included in the analysis are: “MSK-IMPACT sequencing of 720 Melanoma tumor samples with matched normals”; “TCGA Skin Cutaneous Melanoma Source data from GDAC Firehose”; and “Breast Invasive Carcinoma Source data from GDAC Firehose” (Cerami et al, 2012; Gao et al, 2013).

### qPCR UV interference assay

IMR-90 RB1 KO and wild-type cells were grown to confluency and exposed to indicated doses of UV. Cells were immediately lysed in 1% SDS buffer and DNA purified using Quick-DNA Miniprep Kit (D3024; Zymo Research). *GAPDH* was used as DNA loading control in qPCR assays. The percentage inhibition was calculated by first subtracting the Cq value for *TERT* from that of *GAPDH* within each −UV and +UV sample and cell line. The resulting −UV and +UV values were then subtracted from each other to calculate X, which was used in (1 − (1/2^X)) × 100 to calculate percent inhibition. *MECOM* primer sequences are: forward 5′-AGCAGGTCTTGATTCGACGTT-3′; reverse 5′-CACAGGTGAGGTCTGCCATA-3′. *GAPDH* primer sequences are: forward 5′-GACTGAGATTGGCCCGATG-3′; reverse 5′-GACTGAGATTGGCCCGATG-3′. *TERT* primer sequences are: forward 5′-CTGGAAGGTGAAGGGGCAG-3′; reverse 5′-GGGCTCCCAGTGGATTCG-3′.

### Telomere length quantification

Telomere length estimation was carried out using two separate programs: Teltale from St. Jude children’s research hospital (github: https://stjude.github.io/teltale/) and Telomere Hunter (Feuerbach et al, 2019). ChIP-Seq input from both RB1 KO and wild-type were aligned to hg19, and the resulting bam file was used as the input for both programs. Teltale counts the number of reads in the file consisting of seven or more consecutive occurrences of the telomere motif TTAGGG or its reverse complement. Telomere Hunter estimates the telomere content by counting telomeric reads and normalizing the count with the number of reads with comparable GC content. Unlike Teltale, Telomere Hunter also includes telomere variant repeats for analysis. The analysis was followed with telomere qPCR, as previously described (Cawthon, 2002). The primer sequences were: telo fwd, 5′-CGGTTTGTTTGGGTTTGGGTTTGGGTTTGGGTTTGGGTT-3′; and telo rev, 5′-GGCTTGCCTTACCCTTACCCTTACCCTTACCCTTACCCT-3′; 36B4 fwd, 5′-AGCAAGTGGGAAGGTGTAATCC-3′; and 36B4 rev, 5′-CCCATTCTATCATCAACGGGTACAA-3′. Primer pair teloF/R was used to estimate telomere content, and 36B4F/R was used as control.

### XR-seq repair analysis

CPD nucleotide excision repair rates were generated using eXcision Repair-sequencing (XR-seq) and downloaded from Gene Expression Omnibus accession GSE76391 (Adar et al, 2016). Briefly, normal human fibroblasts (NHF1) were irradiated with UVC and were collected 1, 4, 8, 16, 24, and 48 h after irradiation. TFIIH bounded chromatin containing CPD lesions were then immunoprecipitated with anti-TFIIH antibody, followed by IP with anti-CPD antibody. Replicates and strand-specific signals for each time point were averaged for analysis. Cumulative signals for each time point were calculated by summation with all previous time points.

## Data Availability

Data sets produced in this study are available on the Gene Expression Omnibus database under: GSE173125, GSE173126, GSE173127, and GSE173128. Computational scripts can be found at https://github.com/orgs/MorrisonLabSU/.

## Supplementary Material

Reviewer comments
